# The AKT inhibitor, MK-2206, attenuates ABCG2-mediated drug resistance in lung and colon cancer cells

**DOI:** 10.3389/fphar.2023.1235285

**Published:** 2023-07-13

**Authors:** Hai-Ling Gao, Qingbin Cui, Jing-Quan Wang, Charles R. Ashby, Yanchun Chen, Zhi-Xin Shen, Zhe-Sheng Chen

**Affiliations:** ^1^ Department of Histology and Embryology, Weifang Medical University, Weifang, Shandong, China; ^2^ Department of Pharmaceutical Sciences, College of Pharmacy and Health Sciences, St. John’s University, Queens, NY, United States; ^3^ Affiliated Hospital of Weifang Medical University, Weifang, Shandong, China

**Keywords:** cancer, MDR, ABCG2, MK-2206, sensitization

## Abstract

**Introduction:** The overexpression of ATP-binding cassette (ABC) transporters, ABCB1 and ABCG2, are two of the major mediators of multidrug resistance (MDR) in cancers. Although multiple ABCB1 and ABCG2 inhibitors have been developed and some have undergone evaluation in clinical trials, none have been clinically approved. The compound, MK-2206, an inhibitor of the protein kinases AKT1/2/3, is undergoing evaluation in multiple clinical trials for the treatment of certain types of cancers, including those resistant to erlotinib. In this *in vitro* study, we conducted *in vitro* experiments to determine if MK-2206 attenuates multidrug resistance in cancer cells overexpressing the ABCB1 or ABCG2 transporter.

**Methodology:** The efficacy of MK-2206 (0.03–1 μM), in combination with the ABCB1 transporter sub-strates doxorubicin and paclitaxel, and ABCG2 transporter substrates mitoxantrone, SN-38 and topotecan, were determined in the cancer cell lines, KB-C2 and SW620/Ad300, which overexpress the ABCB1 transporter or H460/MX20 and S1-M1-80, which overexpress the ABCG2 transporter, respectively. The expression level and the localization of ABCG2 transporter on the cancer cells membranes were determined using western blot and immunofluorescence assays, respectively, following the incubation of cells with MK-2206. Finally, the interaction between MK-2206 and human ABCG2 transporter was predicted using computer-aided molecular modeling.

**Results:** MK-2206 significantly increased the efficacy of anticancer compounds that were substrates for the ABCG2 but not the ABCB1 transporter. MK-2206 alone (0.03–1 μM) did not significantly alter the viability of H460/MX20 and S1-M1-80 cancer cells, which overexpress the ABCG2 transporter, compared to cells incubated with vehicle. However, MK-2206 (0.3 and 1 μM) significantly increased the anticancer efficacy of mitoxantrone, SN-38 and topotecan, in H460/MX20 and S1-M1-80 cancer cells, as indicated by a significant decrease in their IC50 values, compared to cells incubated with vehicle. MK-2206 significantly increased the basal activity of the ABCG2 ATPase (EC50 = 0.46 μM) but did not significantly alter its expression level and sub-localization in the membrane. The molecular modeling results suggested that MK-2206 binds to the active pocket of the ABCG2 transporter, by a hydrogen bond, hydrophobic interactions and π-π stacking.

**Conclusion:** These *in vitro* data indicated that MK-2206 surmounts resistance to mitoxantrone, SN-38 and topotecan in cancer cells overexpressing the ABCG2 transporter. If these results can be translated to humans, it is possible that MK-2206 could be used to surmount MDR in cancer cells overexpressing the ABCG2 transporter.

## Introduction

Although significant progress has been made in developing novel anticancer drugs, efficacious cancer treatments, including chemotherapeutic and radio-therapeutic approaches, can be decreased or abrogated by the development of multidrug resistance (MDR), which is responsible for producing a significant proportion of cancer-related deaths (Vasan et al., 2019; Yang et al., 2020a; Cui et al., 2022). Numerous studies have shown that MDR can be mediated by: 1) an overexpression of ATP-binding cassette (ABC) transporter; 2) mutations in the cellular drug target; 3) increasing in the metabolism of drugs to metabolites that are efficacious or inefficacious; 4) altering the tumor microenvironment to increase the probability of cancer cell survival; 5) an increase in the repair of damaged DNA; 6) a decrease or inhibition of cell death mechanisms; 7) sequestration of drugs in cellular compartments (i.e., lysosomes) and 8) evasion of the host immune response (Zhitomirsky and Assaraf, 2016; Rathore et al., 2017; Kaur et al., 2020; Li et al., 2020; Wang et al., 2021a; Wang et al., 2021b; Ni et al., 2021; Meng et al., 2022).

ABC transporters are a family of proteins, primarily located on the cellular membrane, which efflux toxic endogenous and xenobiotic molecules from cells (Chen, 2011; Chen and Tiwari, 2011; Kathawala et al., 2015; Li et al., 2016). Numerous studies indicate that certain types of cancer cells overexpress the ABCB1 [also known as P-glycoprotein (P-gp)] or ABCG2 transporter [also known as breast cancer resistanceprotein (BCRP)], which significantly decreases the intracellular concentration of anticancer drugs, thereby decreasing or abrogating their efficacies, conferring resistance to these drugs (Engle and Kumar, 2022; Moinul et al., 2022). Furthermore, both ABCB1 and ABCG2 transporters can decrease the efficacy of anticancer drugs that are structurally and mechanistically distinct, producing MDR (Wei et al., 2020; Sucha et al., 2022). Although various inhibitors/modulators of the ABCB1 and ABCG2 transporters have been well characterized, none of these compounds have been approved for cancer, due to limited clinical benefits, toxic effects and problematic adverse drug-drug interactions (Pena-Solorzano et al., 2017; De Vera et al., 2019; Dong et al., 2020; Kukal et al., 2021; Liu et al., 2022; Moinul et al., 2022). Recently, drug repurposing has emerged as a feasible strategy to overcome ABCB1- or ABCG2-mediated drug resistance in cancer (Wang et al., 2020a; Zhang et al., 2020; Wu et al., 2022).

**FIGURE 1:**
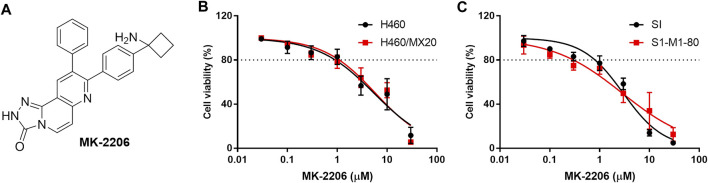
The effect of MK-2206 on the viability of the parental H460 and S1 (non-resistant) and the H460/MX20 and S1-M1-80 (MDR) cancer cells. **(A)** The structure of MK-2206. The effect of MK-2206 on the viability of H460 and H460/MX20 cancer cells **(B)** and **(C)** the effect of MK-2206 on the viability S1 and S1-M1-80 cancer cells. Each point represents the mean ± SD (n = 3). Percent cell viability was compared to vehicle.

MK-2206 (Figure 1A) has been reported to be an allosteric AKT inhibitor that inhibits AKT1 (IC_50_ = 5 nM), AKT2 (IC_50_ = 12 nM) and AKT3 (IC_50_ = 65 nM) (Hirai et al., 2010; Stottrup et al., 2016). This drug has efficacy in clinical trials for the treatment of breast cancer in patients with epidermal growth factor receptor 2-positive (EGFR+), hormone receptor-negative (HR–), phosphatidylinositol-4,5-bisphosphate 3-kinase catalytic (PI3KCA) or AKT mutations and/or Phosphatase and Tensin Homolog deleted on Chromosome 10 (PTEN) loss/PTEN mutations (Xing et al., 2019; Chien et al., 2020). MK-2206 is also undergoing evaluation in patients diagnosed with refractory lymphoma (Oki et al., 2015), uterine serous carcinoma (Stover et al., 2022), advanced pancreatic cancer and advanced non-small cell lung cancer (Lara et al., 2015). Furthermore, MK-2206 produces synergistic anticancer efficacy when combined with gemcitabine (Wang et al., 2020b), cisplatin and olaparib (Savaee et al., 2022), salinomycin (Savaee et al., 2022) and ruxolitinib (Guvenir and Eroglu, 2022), among others, suggesting that it increases the efficacy of certain anticancer drugs, i.e., it is a chemo-sensitizing drug.

Thus, based on the above information, we conducted *in vitro* experiments and the results indicated that MK-2206 significantly increased the efficacy of the ABCG2 transporter substrates, mitoxantrone, SN-38 and topotecan, in H460/MX20 and S1-M1-80 cancer cells, which overexpress the ABCG2 transporter. In contrast, MK-2206 did not significantly alter the efficacy of the ABCB1 substrates, doxorubicin and paclitaxel, in KB-C2 and SW620/Ad300 cells, which overexpress the ABCB1 transporter (Shi et al., 2007; Dai et al., 2008). We further determined the effect of MK-2206 on the expression levels of the ABCG2 transporter and the localization of the ABCG2 transporter in cancer cells, using Western blot and immunofluorescence assay, respectively. Finally, we used a human homology model of the ABCG2 transporter to assess the interaction of MK-2206 with this protein.

## Materials and methods

### Cell viability assay

The effect of MK-2206 alone (0.03–30 μM) and in combination (0.3 and 1 μM) with mitoxantrone (0.1 nM-10 μM), SN-38 (0.1 nM-10 μM) and topotecan (0.1 nM-10 μM) on the viability of parental NCI-H460 (H460) and ABCG2-overexpressing NCI-H460/MX20 (H460/MX20) lung cancer cells, and parental S1 and ABCG2-overexpressing S1-M1-80 colon cancer cells, were determined using the MTT assay. Similarly, the effect of MK-2206 alone (0.03–30 μM) and in combination (0.3 and 1 μM) with doxorubicin (0.1 nM-10 μM) or paclitaxel (0.1 nM-10 μM), on the viability of parental KB-3-1 and ABCB1-overexpressing KB-C2 human epidermoid carcinoma cells, and parental SW620 and ABCB1-overexpressing SW620/Ad300 colorectal cancer cells, were also determined using the MTT assay. Ko-143 (3 μM) or verapamil (3 μM) were used as positive controls, as these compounds inhibit the ABCG2 or ABCB1 transporter, respectively (Allen et al., 2002; Laberge et al., 2014). Briefly, cancer cells were seeded at a density of 3,000-5,000/well in 96-well plate and incubated overnight. Subsequently, the corresponding test drug was added into each well and the cells were cultured for 72 h. Next, 20 μL of the MTT solution (5 mg/mL) was added to the wells and the cells were cultured for 4 h, and the medium was carefully removed and then 100 μL of DMSO was added and the absorption was recorded at a wavelength of 490 nm, using a microplate absorbance reader. The viability of each cell line was plotted and IC_50_ values were calculated using GraphPad Prism 7.00 (GraphPad, San Diego, CA, United States).

### Western blot assay

The expression levels of ABCG2 in the H460 or H460/MX20 cancer cells after incubation with MK-2206 were determined using the Western blot assay as previously described (Wang J. et al., 2020). Briefly, H460/MX20 cancer cells were seeded at a density of 1,000,000/well in a 6-well plate and cultured overnight. MK-2206 (0.1, 0.3, and 1 μM) and the cells were incubated for 24 h or with MK-2206, at 1 μM, for 24, 48 and 72 h. Subsequently, the cells were washed three times with PBS and lysed in a modified RIPA buffer (150 mM NaCl, 1 mM EDTA, 50 mM Tris, 1% Triton X-100, 0.1% SDS, 0.5% sodium deoxycholate), with a phosphatase inhibitor cocktail (Roche, Indianapolis, IN, United States) for 15 min on ice. The cell debris was removed by centrifugation at 12,000 g for 15 min at 4°C. The protein concentration in the lysates was determined using the bicinchoninic acid (BCA) protein assay (ThermoFisher, Rockford, IL, United States). An equivalent amount (20 μg) of protein was subjected to SDS-PAGE (constant voltage at 120V), followed by transfer to PVDF membranes. An ABCG2 antibody (Catalog number MAB4146, Millipore) and control GAPDH (Catalog number MA5-15738, Millipore) were used to identify ABCG2 or GAPDH, respectively. H460 parental cells were used as the negative control as they do not overexpress the ABCG2 transporter (Henrich et al., 2006). The Enhanced Chemiluminescence (ECL) kit (Thermo Fisher Scientific Inc., Waltham, MA, United States) was used and the signal was captured by X-ray film. The analysis of protein expression levels was performed using ImageJ (NIH, MD, United States).

### Immunofluorescence assay

The effect of MK-2206 on the localization of ABCG2 was determined using a previously reported immunofluorescence technique (Ji et al., 2019). Briefly, 30,000/well cells were seeded in a 12-well plate and cultured overnight. Subsequently, the cells were incubated with MK-2206 (1 μM) for 24 h. Next, the medium was removed and the cells were washed three times with PBS. The cells were incubated with 4% polyformaldehyde and 0.25% Triton X-100 for 5 min. Cells were cultured with an ABCG2 antibody (Catalog number MAB4146, Millipore) over night at 4°C. After removal of the ABCG2 antibody by gently washing with cold PBS three times, the cells were co-cultured with fluorescent IgG antibody (Catalog number 7076S, CTS) for 2 h. The nucleus was stained with propidium iodide (PI). The images of the cells were taken using a Nikon TE-2000S fluorescence microscopy (Nikon Instruments Inc., Melville, NY), at an excitation wavelength of 535 nm to visualize the nuclei and a wavelength of 395 nm to visualize the ABCG2 transporter (×200 magnification) (Ji et al., 2019). Two pictures were taken for the same set of cells, and one merged figure was generated by overlapping the images for the ABCG2 transporter and the nuclei, using Photoshop (CS6, Adobe).

### ATPase assay

The ATPase activity of vanadate-sensitive ABCG2 was determined as previously described (Ji et al., 2018; Yang et al., 2020b; Narayanan et al., 2021a). High Five insect cells was used to prepare ABCG2 membrane vesicles. Ten μg of the membranes were incubated in an assay buffer containing 50 mM of 2-(N-morpholino) ethane sulfonic acid (MES, pH 6.8), 50 mM of KCl, 5 mM of sodium azide, 2 mM of EGTA, 2 mM of dithiothreitol (DTT), 1 mM of ouabain, and 10 mM of MgCl_2_ for 5 min. Subsequently, MK-2206 (0.1–20 μM) was incubated with membrane vesicles for 3 min. ATP hydrolysis was initiated by adding 5 mM of Mg-ATP and then after incubation at 37°C for 20 min, the reaction was terminated by adding 100 μL of a 5% SDS solution. The level of inorganic phosphate (Pi) was determined by measuring the absorption at 880 nm, using an Accuskan GO UV/Vis Microplate Spectrophotometer (Thermo Fisher Scientific Inc., Waltham, MA, United States).

### Molecular docking of MK-2206 with human ABCG2 model

The three dimensional structure of MK-2206 was constructed for docking simulation with a human ABCG2 homology model as previously described (Wang L Q et al., 2019). The human ABCG2 protein model, 6ETI, was obtained from the Research Collaboratory for Structural Bioinformatics (RCSB) Protein Data Bank (PDB). The protein model was constructed from an inward-facing Ko143-bound human ABCG2 crystal structure, at a resolution of 3.1 Å (Jackson et al., 2018). Docking calculations were performed using the program, AutoDock Vina (version 1.1.2), in the default method (Wang L et al., 2019). Hydrogen atoms and partial charges were added using AutoDockTools (ADT, version 1.5.4). Docking grid center coordinates were determined using the bound ligand, Ko143, provided in the 6ETI PDB files. Receptor/ligand preparation and docking simulation were performed using default settings. The top-scoring pose (based on the affinity score in kcal/mol) was selected for further analysis and visualization.

### Statistics

Data were expressed as mean ± SD from at least three independent experiments and analyzed using GraphPad Prism 7.00 software (GraphPad, San Diego, CA, United States). The effects of MK-2206 on the efficacy of SN-38, mitoxantrone and topotecan in the cancer cell lines was determined using a one-way ANOVA, followed by a *post hoc* analysis using the Newman-Keuls test. The effects of MK-2206 on the ABCG2 expression level in H460 and H460/MX20 cell lines were analyzed by Student’s t-test after quantification of the bands by ImageJ (NIH, MD, United States). The *a priori* significance value was *p* < 0.05.

## Results

### MK-2206 increases the efficacy of mitoxantrone, SN-38 and topotecan in H460/MX20 and S1-M1-80 cancer cells overexpressing the ABCG2 transporter

The *in vitro* viability of the parental (non-resistant) H460 and S1 and the MDR H460/MX20 and S1-M1-80 cancer cells was decreased by 20% at 1 μM of MK-2206 and the IC_50_ values ranged from 3 to 10 μM (Figures 1B,C). Based on these results, we determined the concentrations of 0.3 and 1 μM of MK-2206 can be used to combine with mitoxantrone, SN-38 and topotecan, which are ABCG2 substrates (Toyoda et al., 2019). Similarly, the cytotoxicity of MK-2206 in parental KB-3-1 and SW620 cells and the MDR KB-C2 and SW620/Ad300 cells, were also determined, and 0.3 and 1 μM of MK-2206 did not significantly affect cell viability (data not shown). The combination of MK-2206 (0.3 and 1 μM) with the anticancer drugs, doxorubicin and paclitaxel, which are substrates for the ABCB1 transporter, did not significantly alter the efficacy of these compounds in cancer cells overexpressing the ABCB1 transporter (data not shown). Thus, we next conducted experiments to determine the effect of MK-2206 on the efficacy of drugs in cancer cells overexpressing the ABCG2 transporter.

#### The effect of MK-2206 on the efficacy of mitoxantrone in the parental and MDR cancer cell lines

**FIGURE 2:**
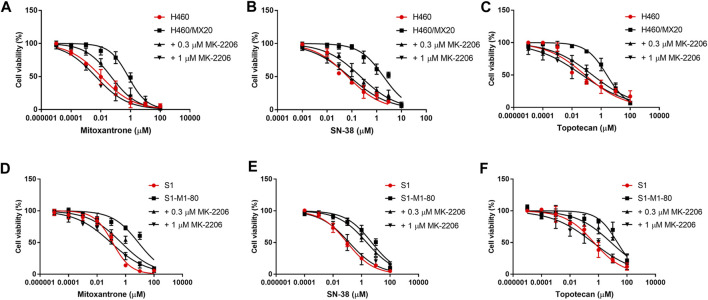
The effect of MK-2206 on the efficacy of mitoxantrone, SN-38 and topotecan in the ABCG2-overexpressing cancer cell lines, H460/MX20 **(A, B, C)** and S1-M1-80 **(D, E, F)**. Each point in the graphs represents the % mean cell viability ± SD (n = 3).

The concentration response curves of mitoxantrone in the H460/MX20 and S1-M1-80 cancer cells were significantly shifted to the right of the curve of the non-resistant parental H460 and S1 cancer cells, respectively (Table 1; Figures 2A,D). As previously reported (Fan et al., 2018; Wu et al., 2022), the IC_50_ value for Mitoxantrone in the H460/MX20 MDR lung cancer cells incubated with was significantly greater (66.3-fold) than the IC_50_ value for the H460 parenteral lung cancer cells, which do not over express the ABCG2 transporter, i.e., non-drug resistant (Table 1; Figures 2A,D). Similarly, the IC_50_ value of mitoxantrone for the MDR S1-M1-80 colon cancer cells incubated with vehicle was also significantly greater (64.2 fold) than the S1 parental cells, which are non-drug resistance (Table 1; Figures 2A,D). In contrast, the incubation of H460/MX20 and S1-M1-80 MDR cancer cells with 0.3 μM of MK-2206 significantly decreased the IC_50_ values by 14.6 and 16-fold, respectively, compared to cells incubated with vehicle (Table 1; Figures 2A,D). Furthermore, the incubation H460/MX20 and S1-M1-80 MDR cancer cells with 1 μM of MK-2206 significantly decreased the IC_50_ values by 78- and 86-fold, respectively, compared to cells incubated with vehicle (Table 1; Figures 2A,D). It is important to note that at 1 μM, MK-2206 decreased the IC_50_ value for the MDR H460/MX20 and S1-M1-80 cancer cells below those for the parental H460 and S1 cancer cells, respectively.

**TABLE 1:** The effect of MK-2206 on the efficacy of mitoxantrone, SN-38 and topotecan in cancer cells overexpressing the ABCG2 transporter.

Treatments	IC_50_ ± SD (μM)^$^			
	H460	H460/MX20	S1	S1-M1-80
Mitoxantrone + vehicle	0.0106 ± 0.008	0.703 ± 0.374^###^	0.123 ± 0.023	7.901 ± 0.917^###^
+ 0.3 μM MK-2206		0.048 ± 0.022^***^		0.493 ± 0.180^***^
+ 1 μM MK-2206		0.009 ± 0.002^***^		0.092 ± 0.022^***^
+ 3 μM Ko-143		0.059 ± 0.025^***^		0.113 ± 0.136^***^
SN-38 + vehicle	0.069 ± 0.017	1.917 ± 0.562^###^	0.097 ± 0.023	5.187 ± 2.722^###^
+ 0.3 μM MK-2206		0.225 ± 0.042^***^		2.309 ± 0.341^**^
+ 1 μM MK-2206		0.081 ± 0.021^***^		0.136 ± 0.131^***^
+ 3 μM Ko-143		0.147 ± 0.095^***^		0.096 ± 0.025^***^
Topotecan + vehicle	0.081 ± 0.041	2.498 ± 0.043^###^	0.481 ± 0.122	21.46 ± 9.603^##^
+ 0.3 μM MK-2206		0.257 ± 0.054^***^		7.663 ± 6.434^*^
+ 1 μM MK-2206		0.051 ± 0.049^***^		0.407 ± 0.441^**^
+ 3 μM Ko-143		0.077 ± 0.071^**^		0.591 ± 0.176^**^

^$^ Each value represents the mean IC50 value **±** the S.D. **(c**alculated by GraphPad 7.00), based on three independent experiments.

^##^, ^###^, *p* < 0.01, 0.001 vs. anticancer drug + vehicle in H460 or S1 cell lines; ^*^, ^**^, ^***^, *p* < 0.05, 0.01, 0.001 vs. anticancer drug + vehicle in H460/MX20 or S1-M1-80 cell lines.

#### The effect of MK-2206 on the efficacy of SN-38 in the parental and MDR cancer cell lines

As shown in Figures 2B,E and Table 1, H460/MX20 and S1-M1-80 cancer cells were resistant to SN-38, compared to the non-resistant parental H460 and S1 cancer cells, respectively. The IC_50_ value for SN-38 in the H460/MX20 MDR lung cancer cells incubated with SN-38 was 28-fold greater than the IC_50_ value for the H460 parenteral lung cancer cells (Table 1; Figures 2A,D). Similarly, the IC_50_ value of SN-38 for the MDR S1-M1-80 colon cancer cells incubated with vehicle was also significantly greater (53.5-fold) than the non-drug resistant S1 parental cells (Table 1; Figures 2B,E). In contrast, the incubation of H460/MX20 and S1-M1-80 MDR cancer cells with 0.3 μM of MK-2206 significantly decreased the IC_50_ values by 8.5 and 2.2-fold, respectively (Table 1; Figures 2B,E). Furthermore, the incubation H460/MX20 and S1-M1-80 MDR cancer cells with 1 μM of MK-2206 significantly decreased the IC_50_ values by 23.7- and 38.1-fold, respectively, compared to cells incubated with vehicle (Table 1; Figures 2B,E). It should be noted that MK-2206, which fully restored the efficacy of mitoxantrone, did not fully restore the efficacy of SN-38 in MDR H460/MX20 and S1-M1-80 cancer cells to the level of that of the parental H460 and S1 cancer cells.

#### The effect of MK-2206 on the efficacy of topotecan in the parental and MDR cancer cell lines.

The results in Figures 2C,F and Table 1 indicated that H460/MX20 and S1-M1-80 cancer cells were resistant to topotecan. The IC_50_ value for topotecan in the H460/MX20 MDR lung cancer cells incubated with topotecan was significantly greater (30.8-fold) than the IC_50_ value for the H460 parenteral lung cancer cells (Table 1; Figures 2C,F). Similarly, the IC_50_ value of topotecan for the MDR S1-M1-80 colon cancer cells incubated with vehicle was also significantly greater (44.6-fold) than the non-resistant S1 parental cells (Table 1; Figures 2C,F). In contrast, the incubation of H460/MX20 and S1-M1-80 MDR cancer cells with 0.3 μM of MK-2206 significantly decreased the IC_50_ values of topotecan by 9.7 and 2.8-fold, respectively, compared to cells incubated with vehicle (Table 1; Figures 2C,F). Furthermore, the incubation H460/MX20 and S1-M1-80 MDR cancer cells with 1 μM of MK-2206 significantly decreased the IC_50_ values by 49- and 53-fold, respectively, compared to cells incubated with vehicle (Table 1; Figures 2C,F). MK-2206 decreased the IC_50_ value of topotecan for the MDR H460/MX20 and S1-M1-80 cancer cells below those for the parental H460 and S1 cancer cells, respectively, a finding similar to that for mitoxantrone.

It should be noted that 1 μM of MK-2206 produced an increase in the efficacy of mitoxantrone, SN-38 and topotecan in the H460/MX20 and S1-M1-80 cancer cells, which overexpress the ABXCG2 transporter, was greater than that of Ko-143, a compound that inhibits the ABCG2 transporter (Allen et al., 2002).

### The effect of MK-2206 on the expression level of the ABCG2 protein in H460/MX20 cancer cells

**FIGURE 3:**
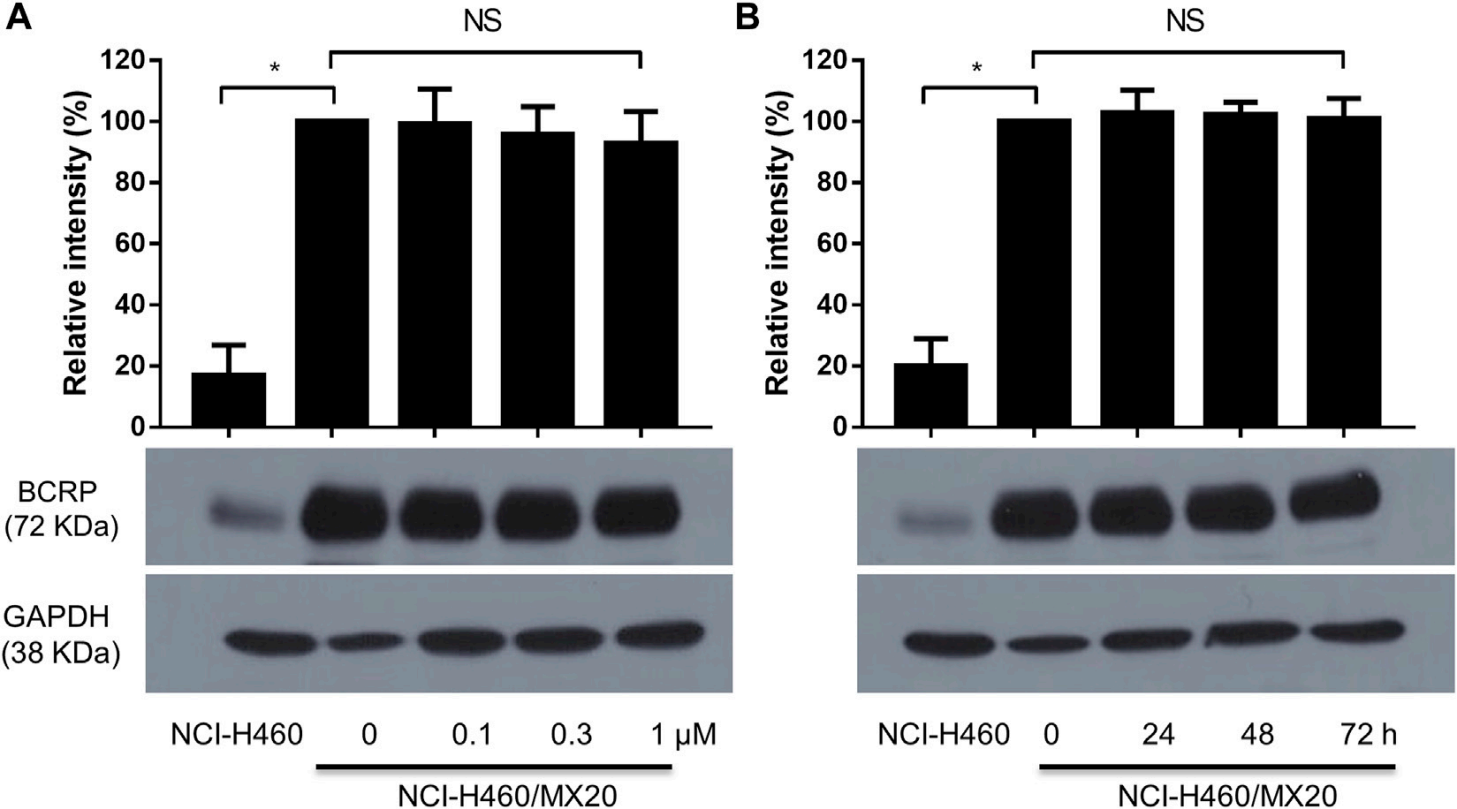
**(A)** The effect of the incubation of H460/MX20 cancer cells for 24 h with vehicle (0), 0.1, 0.3 or 1 μM of MK-2206. **(B)** The effect of the incubation of H460/MX20 cancer cells with vehicle (0) or 1 μM of MK-2206, for 24, 48 or 72 h. GAPDH was used as the control. Each value represents the mean ± the SD (n = 3).

It is possible that MK-2206 could have increased the efficacy of the anticancer drugs by decreasing the levels of ABCG2 protein. Therefore, we determined the effect of MK-2206 on the expression of ABCG2 in H460/MX20 cells. The incubation of cells with either 0.1, 0.3 or 1 μM of MK-2206 for 24 h did not significantly alter the expression of ABCG2, compared to cells incubated with vehicle (Figure 3A). Furthermore, the incubation of H460/MX20 cells with 1 μM of MK-2206 for 24, 48 or 72 h, did not significantly alter the expression of ABCG2, compared to cells incubated with vehicle (Figure 3B). Thus, these results suggest that it is unlikely that MK-2206 increased the efficacy of the ABCG2 substrates, mitoxantrone, SN-38 and topotecan by decreasing the expression of ABCG2.

### MK-2206 did not alter the localization of the ABCG2 protein on the cancer cell membrane

**FIGURE 4:**
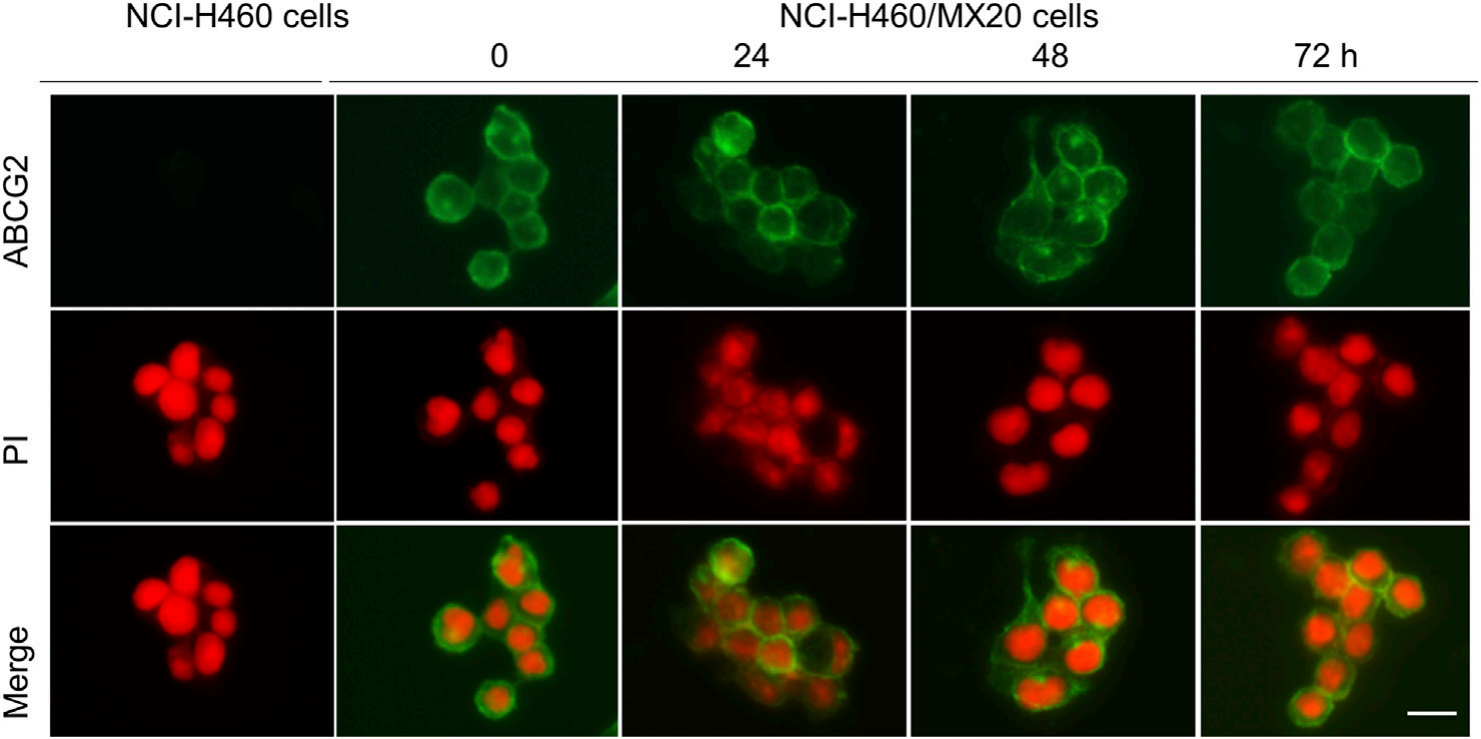
The effect of the incubation of NCI-H460 (drug-sensitive) and NCI-H460/MX20 (drug resistant) cancer cells with 1 μM of MK-2206, for 0, 24, 48 or 72 h, on the intracellular distribution of the ABCG2 transporter, using immunofluorescence. Nuclei were stained with propidium iodide (PI). The merge images represent the combination of an image with PI and ABCG2 were merged into a single image. Scale bar = 25 μm.

The ABCG2 transporter is localized on the plasma cell membrane, where it uses ATP to pump out ABCG2 substrates, such as mitoxantrone, SN-38 and topotecan, producing MDR (Mo and Zhang, 2012). Consequently, we determined if MK-2206 altered the localization of the ABCG2 transporter, as this would abrogate its removal of the above mentioned anticancer drugs. The incubation of H460/MX20 cancer cells with 1 μM of MK-2206 for 24, 48 or 72 h did not significantly alter the localization of the ABCG2 transporter, compared to cells incubated with vehicle (Figure 4). In contrast, the parenteral cell line, NCI-H460 cells, which are non-resistant, do not overexpress ABCG2 transporters (Figure 4). These results suggest that it is unlikely that MK-2206 increases the efficacy of mitoxantrone, SN-38 and topotecan by affecting the cellular localization of the ABCG2 transporter.

### MK-2206 stimulated basal ABCG2 ATPase activity

**FIGURE 5:**
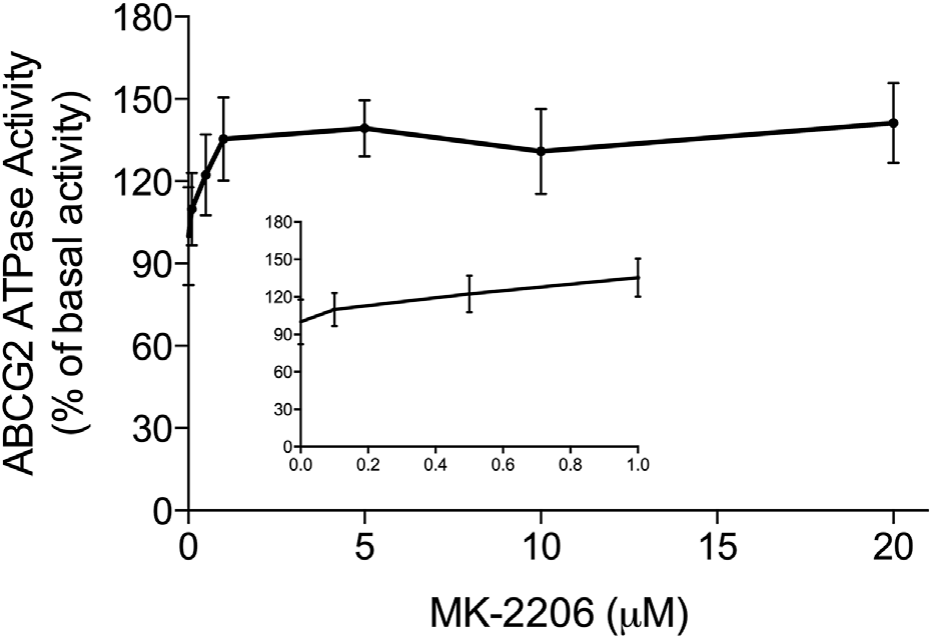
The *in vitro* effect of MK-2206 on the basal activity of ABCG2 ATPase in High-five insect cells. Each point on the graphs represents the mean % change in basal ATPase activity ± SD (n = 3).

The hydrolysis of ATP by the ABCG2 transporter ATPase has been suggested to be linked to the substrate efflux function of the ABCG2 transporter (Eckenstaler and Benndorf, 2020; Kowal et al., 2021). Furthermore, ABCG2 substrates, including the drugs used in this study can increase or decrease the basal activity of the ABCG2 ATPase (Glavinas et al., 2007). Therefore, we conducted *in vitro* experiments to ascertain if MK-2209, at various concentrations (0.1–20 μM), affected the ATPase activity of the ABCG2 transporter. The results indicated that MK-2206 produced a maximal increase of 1.41-fold in the basal activity of the ABCG2 transporter ATPase (Figure 5). The concentration required to increase the basal ABCG2 ATPase activity by 50% (EC_50_) was 0.46 μM. Based on these data, we hypothesized that MK-2206 binds to the drug-substrate site on the ABCG2 transporter, although this remains to be determined.

### MK-2206 interacts with the ABCG2 transporter as determined by docking analysis

**FIGURE 6:**
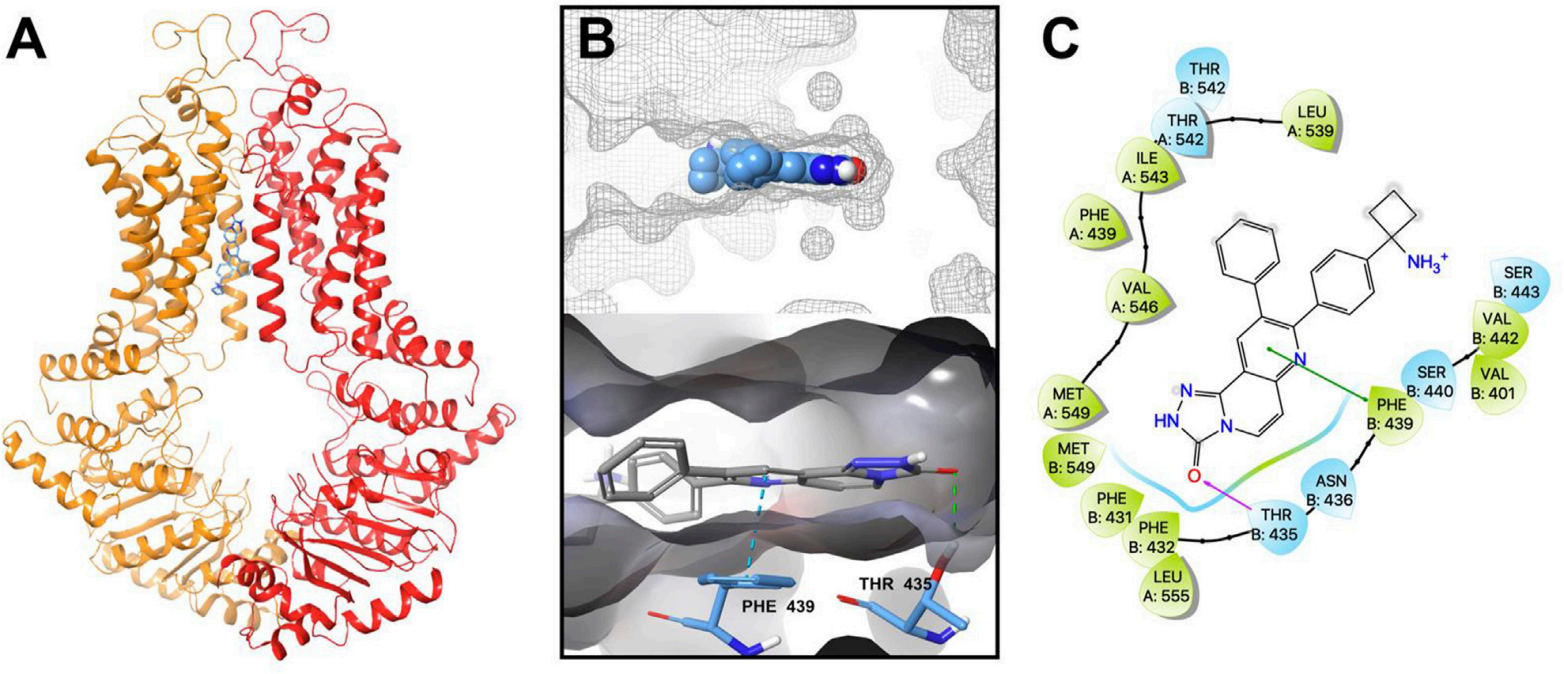
The interaction between MK-2206 and human ABCG2 protein. **(A)** The structure of the best-scoring pose of MK-2206 in the drug binding pocket of ABCG2 protein (6ETI). ABCG2 is displayed as colored tubes and ribbons. MK-2206 is displayed as colored sticks. Carbon: blue; oxygen: red; nitrogen: blue. **(B)** Top: the best-scoring pose of MK-2206 in the drug binding pocket of ABCG2 showing the molecule surface. Bottom: the interactions between MK-2206 and ABCG2 (6ETI) binding pocket. The important residues are displayed as colored sticks (carbon: blue; oxygen: red; nitrogen: blue). MK-2206 is displayed as colored sticks (carbon: grey; oxygen: red; nitrogen: blue). Hydrogen bonds are displayed as green dashed lines. The π-π stacking interactions are displayed as blue dashed lines. **(C)** A two-dimensional diagram of the interaction between MK-2206 and ABCG2. The important amino acids were displayed as colored bubbles (green: hydrophobic; blue: polar). Purple solid lines with arrow indicate hydrogen bonds. Green solid lines without arrows indicate π-π stacking interactions.

Our *in vitro* results indicated that MK-2206 increased the anticancer efficacy of the anticancer drugs, mitoxantrone, SN-38 and topotecan, which are substrates for the ABCG2 transporter. We hypothesized that MK-2206 could significantly increase the efficacy of the anticancer drugs by interacting with the ABCG2 transporter, which could inhibit the efflux of these drugs. Therefore, we used a docking simulation approach in a Ko143-bound human ABCG2 crystal structure model (6ETI) to determine if MK-2206 interacted with the ABCG2 protein. Our results indicated that MK-2206 docked into the Ko143 binding site, with a high docking score of −9.0 kcal/mol. The binding of MK-2206 to the ABCG2 protein was primary due to hydrophobic interactions (Figure 6). MK-2206 was positioned and stabilized in the hydrophobic cavity formed by Phe439, Leu539, Thr542, Ile543, Val546, Met549 and Leu555 in chain A, and Phe431, Phe432, Phe439, Val442 and Ser443 in chain B (Figure 6). MK-2206 binding was stabilized by a hydrogen bond formed between Thr435 and oxygen atom in MK-2206 and a π-π stacking interaction with Phe439 and the pyridine of MK-2206, in chain B.

## Discussion

In this study, our results indicated that MK-2206, at 0.3 and 1 μM, did not significantly decrease the viability of the parental H460 and ABCG2-overexpressing H460/MX20 lung cancer cells and parental S1 and MDR ABCG2-overexpressing S1-M1-80 colon cancer cells. Furthermore, MK-2206 did not significantly alter the efficacy of doxorubicin and paclitaxel, which are substrates for the ABCB1 transporter, in the ABCB1-overexpressing cancer cells, KB-C2 and SW620/Ad300.

Numerous studies have reported that the overexpression of the ABCG2 transporter is one of the major mediators of MDR in cancer cells (Hira and Terada, 2018; Wang et al., 2021a; Kukal et al., 2021). Our results, indicated that mitoxantrone, SN-38 and topotecan were efficacious in the non-resistant parental H460 and S1 cancer cells, whereas resistance occurred to these anticancer drugs in the ABCG2-overexpressing H460/MX20 and S1-M1-80 cancer cells, with a resistant-fold ranging from ∼30 to 70, consistent with our previous findings (Fan et al., 2018; Wu et al., 2022). The *in vitro* incubation of the ABCG2 overexpressing cancer cells lines, H460/MX20 and S1-M1-80, with 0.3 μM of MK-2206 decreased the IC_50_ values of 1) mitoxantrone by 14.6 and 16.0-fold; 2) SN-38 by 8.5 and 2.2-fold and 3) topotecan by 9.7 and 2.8-fold, respectively. Furthermore, 1 μM of MK-2206 completely reversed the resistance to mitoxantrone and topotecan but not SN-38, in the MDR cancer cells. Indeed, the incubation of the ABCG2 overexpressing cancer cells lines with 1 μM of MK-2206 decreased the IC_50_ values (i.e., significantly increased the efficacy) of mitoxantrone, SN-38 and topotecan (78- and 86-fold, 23.7- and 38.1-fold, 49- and 53-fold, respectively) to a magnitude that was not significantly different from the non-resistant parental cell lines.

It is important to note that MDR H460/MX20 and S1-M1-80 cells were generated by incubating these cancer cell lines with mitoxantrone, using a concentration escalation approach over a prolonged period, producing mitoxantrone resistance (Robey et al., 2001), and it is possible that the MDR mechanism may not be due to a single factor, i.e., overexpression of the ABCG2 transporter. It has been reported that other critical factors, such as 1) a decrease in the expression of topoisomerase (the cellular target of mitoxantrone (Harker et al., 1991); 2) mutations in the gene, *ipt1*, which encodes the expression of inositol phosphotransferase that is involved in sphingolipid biosynthesis in cancer cells (Ojini and Gammie, 2015); however, the role of *ipt1* in mediating resistance to mitoxantrone resistance remains to be elucidated; and 3) the overexpression of other ABC transporters, e.g., ABCA2, ABCB1 or ABCC1 (Boonstra et al., 2004; Nieth and Lage, 2005). It is important to note that 1) mitoxantrone, SN-38 and topotecan are all topoisomerases inhibitors and 2) a previous study showed that *in vitro* and *in vivo*, in non-small cell lung cancer NCI-H292 cells, MK-2206 produced a synergistic increase in the antiproliferative efficacy of doxorubicin and camptothecin (Hirai et al., 2010), which are topoisomerase inhibitors and thus, it is also possible that MK-2206 may inhibit the activity of topoisomerases, although this remains to be elucidated. Therefore, future experiments should be done to ascertain the efficacy of the anticancer drugs used in this study, in combination with MK-2206, in cells lines where the resistance is only due to the overexpression of the ABCG2 transporter.

It has been reported that the *in vitro* expression level of the ABCG2 transporter plays a key role in determining drug resistance in cancer cells (Mo et al., 2012). Furthermore, the downregulation of the ABCG2 transporter by certain compounds, such as PD153035 and ponatinib, among others, overcome MDR to mitoxantrone, SN-38, and topotecan, which are ABCG2 substrates (Zhang et al., 2018a). In this study, MK-2206 (0.1, 0.3 and 1 μM for 24 h or 1 μM for 24, 48 and 72 h) did not significantly decrease the expression of the ABCG2 protein in H460/MX20 cells. Therefore, it is unlikely that at the concentrations and incubation times used in this study, MK-2206 is increasing the efficacy of mitoxantrone, SN-38 and topotecan by decreasing the expression of the ABCG2 protein.

Newly synthesized ABCG2 transporters (in the endoplasmic reticulum (ER) surface and undergoes glycosylation in the Golgi apparatus) must be transported to the cell membrane to produce its biological functions (Mozner et al., 2019). Consequently, it is possible that the MK-2206-mediated increase in the efficacy of mitoxantrone, SN-38 and topotecan was due to it preventing the translocation and/or insertion of the ABCG2 transporter into the cell membrane of the H460/MX20 cancer cells. However, our immunofluorescence data indicated that the incubation of H460/MX20 cells with MK-2206 (1 μM for 24, 48 and 72 h) did not significantly alter the localization of ABCG2 transporter. Overall, these data suggest that the MK-2206-mediated increase in the efficacy of mitoxantrone, SN-38 and topotecan was not the result of an alteration in the cellular location of the ABCG2 transporter.

It has been shown that the ABCG2 ATPase activity is required for the active transport of substrates from cancer cells (Ozvegy et al., 2002; Han and Zhang, 2004). Furthermore, certain compounds, such as tepotinib, selonsertib, and VS-4718, among others, have been shown to increase the basal activity of the ABCG2 ATPase (Ji et al., 2018; Ji et al., 2019; Wu et al., 2022). Our results indicated that MK-2206 produced a concentration-dependent increase in the basal activity of the ABCG2 ATPase in High-five insect cells. The stimulation of the ABCG2 ATPase basal activity produced by MK-2206 suggests that it interacts with the substrate-drug binding site and inhibits the binding of other substrates competitively, including anticancer drugs, which would decrease the efflux of these substrates by the ABCG2 transporter. However, additional experiments must be conducted to verify this mechanism. Also, the effect of MK-2206 on the ABCG2 ATPase was determined in High-five insect cells and not in cancer cells and thus, it is possible that the effect of MK-2206 on ABCG2 ATPase in cancer cells may be different from that of the High-five insect cells, although this remains to be determined.

Molecular modeling by computer-aided software, such as AutoDock Vina used in this study, can provide data about the interactions of MK-2206 with the human ABCG2 transporter, which may yield visible conformational and topological interactions between MK-2206 and the ABCG2 transporter and a docking score. MK-2206 was docked at the same site as Ko143, an ABCG2 inhibitor, at the transmembrane domain (TMD), and the molecular modeling suggested that MK-2206 could interact with the human ABCG2 transporter by 1) hydrophobic interactions; 2) a hydrogen bond with Thr435 and 3) a π-π stacking interaction with Phe439. MK-2206 had a docking score of −9.0 kcal/mol, which was higher than venetoclax (−12.1 kcal/mol) or AZ-628 (−12.4 kcal/mol) for the human ABCG2 homology mode, using the same method (Wang et al., 2020a; Wang et al., 2020d), suggesting that MK-2206 has a lower binding affinity for the ABCG2 protein than these drugs. However, it is also possible that MK-2206 may bind to a different domain of the human ABCG2 transporter. Thus, further experiments will be required to validate MK-2206’s binding to the human ABCG2 transporter.

Finally, there are data suggesting that the AKT pathway could 1) directly upregulate ABCG2 activity and expression (Wang L et al., 2019; Liu et al., 2021); 2) decrease lysosomal degradation of the ABCG2 protein (Imai et al., 2012a) or 3) mediate ABCG2 expression by indirect pathway, e.g., PTEN, mTOR, etc. (Huang et al., 2014; Liu et al., 2020). Indeed, LY294002, an inhibitor of AKT, has been reported to block the substrate binding area in the ABCG2 transporter (Imai et al., 2012b). However, unlike MK-2206, which is undergoing evaluation in clinical trials, LY294002 is only used for *in vitro* and *in vivo* experiments. Nevertheless, it is possible that the increase in the efficacy of mitoxantrone, SN-38 and topotecan could be due, in part, to the inhibition of AKT by MK-2206, which is an inhibitor of AKT1/2/3. For example, we have previously reported that the compound VKNG-1 decreased the ABCG2 protein level by decreasing the levels of p-AKT in S1-M1-80 cells (Narayanan et al., 2021b). Overall, we hypothesize that MK-2206 may overcome ABCG2-mediated MDR by multiple mechanisms and further studies will be conducted to identify these mechanisms.

Numerous ABCG2 inhibitors have been identified and tested in cell-based and animal models. However, MK-2206, to our knowledge, is the first reported AKT inhibitor to inhibit the ABCG2 transporter and increase the efficacy of anticancer drugs in cancer cells that overexpressed the ABCG2 transporter. MK-2206 is distinct from certain previously reported ABCG2 inhibitors as it did not significantly downregulate the expression of the ABCG2 transporter, whereas compounds such as PD153035 and ponatinib, significantly decreased the expression of the ABCG2 transporter (Sen et al., 2012; Zhang et al., 2018b). However, it remains to be determined if the mRNA that codes for the ABCG2 transporter protein is altered by concentrations of MK-2206 used in this study. Certain ABCG2 inhibitors have been reported to produce a concentration-dependent inhibition of the ATPase activity of ABCG2, such as venetoclax and sitravatinib (Yang et al., 2020a; Wang et al., 2020b), as well as many other compounds, although there are a number of compounds that can stimulate the ATPase activity of ABC transporters (Zhang et al., 2018a; Zhang et al., 2019), including MK-2206. Clearly, in-depth pharmacological and mechanistic studies are needed to delineate the mechanism of action of MK-2206 at the molecular level.

## Conclusion

*In vitro*, MK-2206 significantly increased the efficacy of the anticancer drugs, mitoxantrone, SN-38 and topotecan, in MDR H460/MX20 lung cancer cells overexpressing the ABCG2 transporter. MK-2206 did not significant alter the levels of the ABCG2 protein or the cellular location of the ABCG2 transporter but it increased the basal activity of the ABCG2 ATPase in High-five insect cells. Docking simulations indicated that MK-2206 binds in the substrate binding pocket area of the ABCG2 transporter. It is likely that MK-2206 increases the efficacy of the anticancer drugs used in this study by decreasing their efflux from the MDR cancer cells; however, this remains to be determined.

## Data Availability

The original contributions presented in the study are included in the article/Supplementary Material, further inquiries can be directed to the corresponding authors.
