# Supplementary figures and images for: The AKT inhibitor, MK-2206, attenuates ABCG2-mediated drug resistance in lung and colon cancer cells
Source: Front Pharmacol. 2023 Jul 13;14:1235285. doi: 10.3389/fphar.2023.1235285 (PMC10373739; doi:10.3389/fphar.2023.1235285)

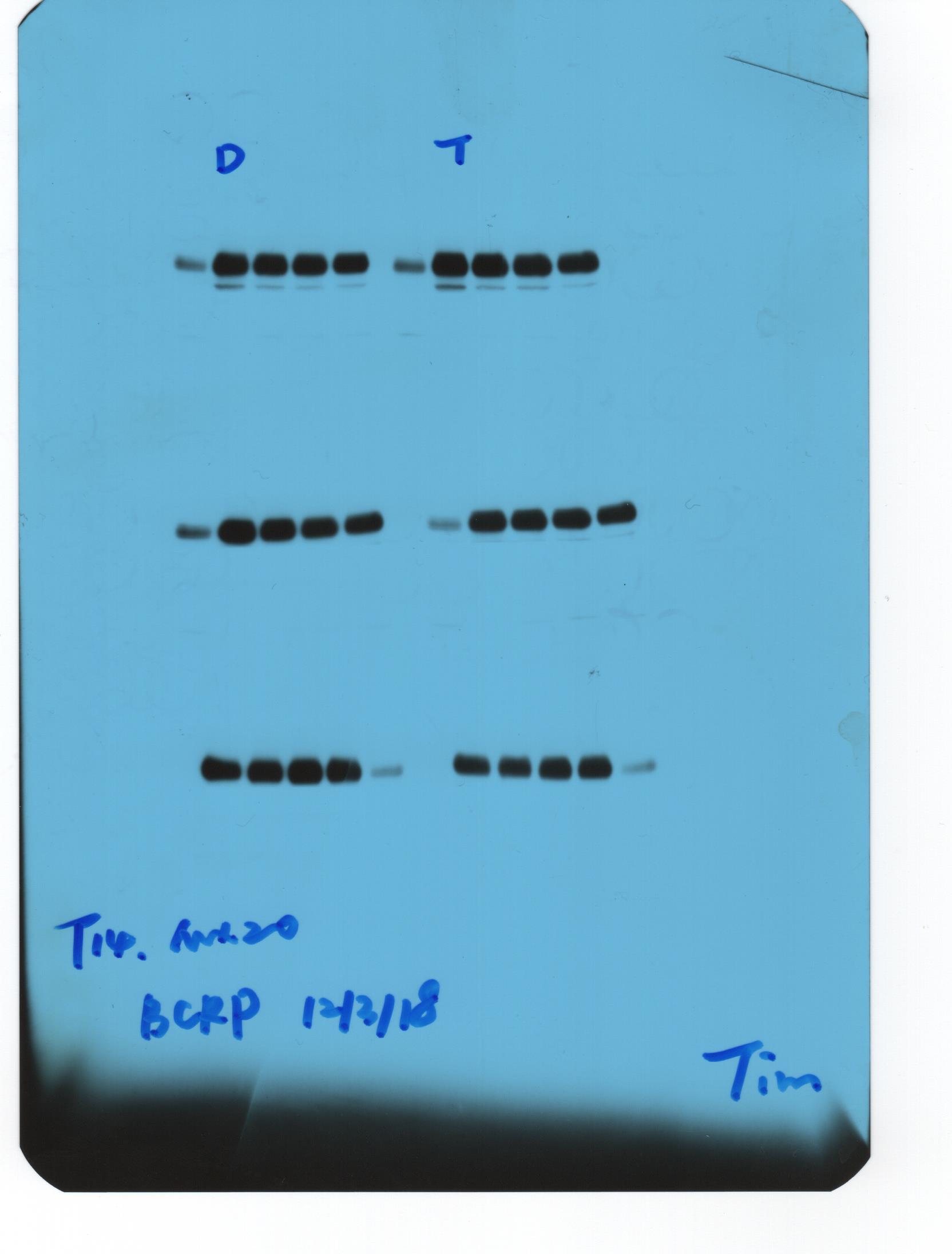

Supplement: Supplementary file 1 [file DataSheet1.ZIP › 120318 T14 MX20 BCRP -1 .jpeg.jpeg]

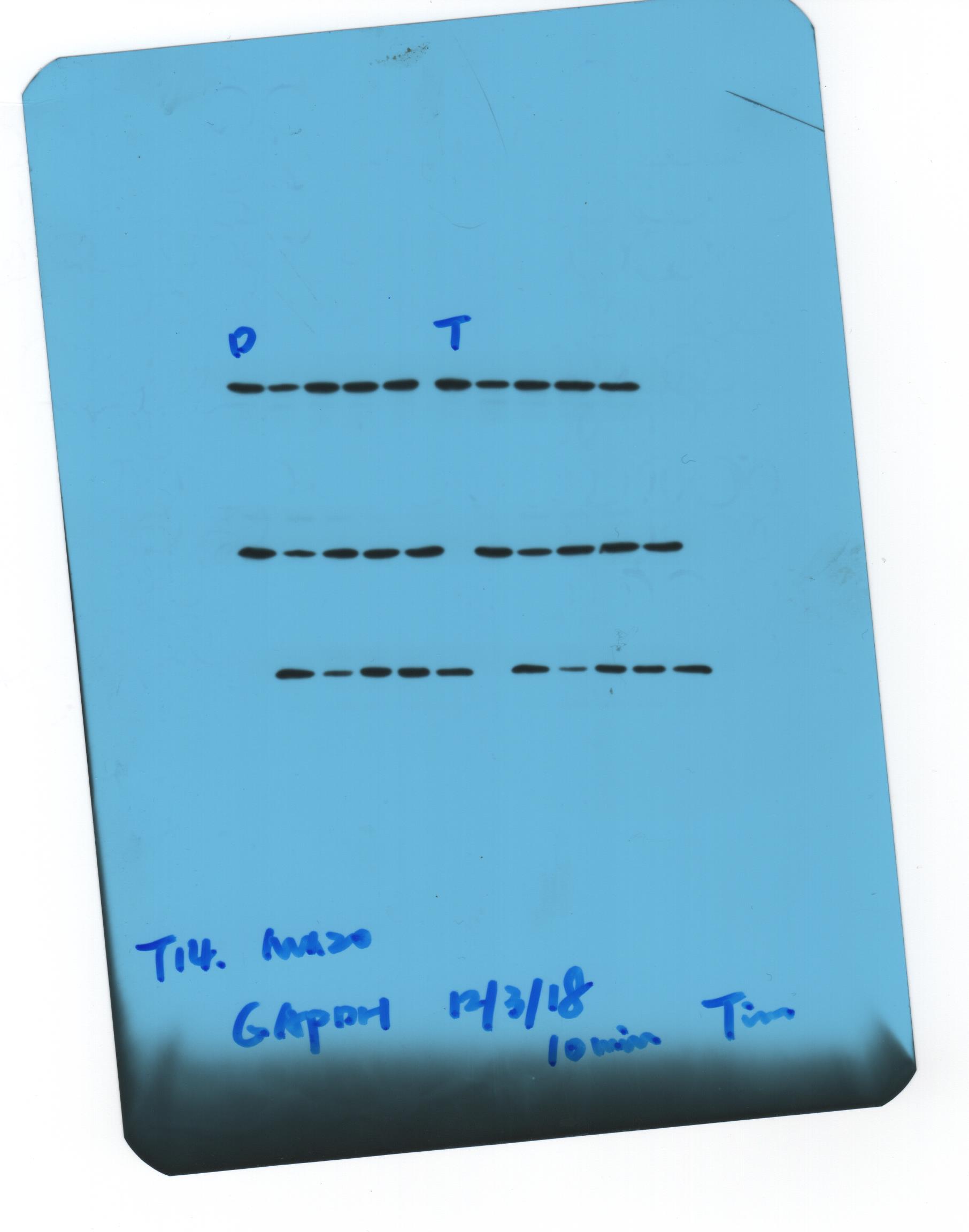

Supplement: Supplementary file 1 [file DataSheet1.ZIP › 120318 T14 MX20 GAPDH 10 min.jpeg]
